# A coupled smoothed particle hydrodynamics-finite volume approach for shock capturing in one-dimension

**DOI:** 10.1016/j.heliyon.2023.e17922

**Published:** 2023-07-05

**Authors:** Conner Myers, Camille Palmer, Todd Palmer

**Affiliations:** School of Nuclear Science and Engineering, Oregon State University, 1791 SW Campus Way, Corvallis, 97331, OR, United States

**Keywords:** Shock capturing, Smoothed particle hydrodynamics, Finite volume method, Sod, Woodward-Colella

## Abstract

A novel approach coupling the finite volume method code Pyro2 and the smoothed particle hydrodynamics code PySPH is introduced and applied to one-dimensional shock problems. The finite volume mesh models the bulk of the system, while regions with discontinuous fluid values are identified and populated with PySPH particles to model the fluid around shocks. The approaches are coupled with boundary cells and ghost particles, with linear interpolation used to extract fluid properties at each timestep in the respective boundary regions. Results from three shock problems using Pyro2, PySPH, and a hybrid approach are presented. The hybrid approach preserves accuracy and computational efficiency in strong shock problems. Additionally, the coupling approach provides a potential avenue for including SPH functionality in FVM simulations, including modeling complex geometries and fluid-structure interaction. The preliminary investigation of the hybrid approach in one dimension highlights the potential efficiency gains of applying coupled FVM-SPH methods to larger blast simulations in two and three dimensions.

## Highlights

• A novel FV-SPH approach is introduced and applied to 1D shock problems.
• SPH particles model the fluid around shocks while FV cells model the bulk fluid.
• Linear interpolation couples fluid properties across FVM and SPH zones.
• The hybrid approach accurately models strong shock simulations while preserving computational efficiency.

## 1 Introduction

Shockwaves, present in many natural and artificial phenomena, are central to the mechanics of processes in industry, defense, and other important fields. The predictive capability of models used to describe shockwaves is vital to understanding the relevant physics and, in many cases, pertinent to the safety of industrial processes. Shockwaves propagate through a medium supersonically and are characterized by a significant jump in fluid properties such as pressure, density, and internal energy. The discontinuous nature of shockwaves makes analytical treatments difficult in simple cases and impossible in most applications.

The difficulty of modeling shockwaves arises from the extreme gradients across fluid properties. Computational methods of simulating fluid or solid media typically involve spatial discretization of the problem and solving the relevant equations at the each point. The medium can be discretized in several ways, including using meshes, cells, particles, abstract mathematical elements, or spectra. In each discretization method, a fine resolution must be taken around the shockwave to accurately resolve the shockwave features, either in space or, in the case of spectral methods, the number of approximation terms.

Limiting the scope of discussion to grid- or cell-based methods, the fine resolution leads to a small timestep via the Courant-Friedrichs-Lewy (CFL) condition, which dictates the necessary timestep for the stability of a solution [1]:

(1) $$\Delta t\leq \frac{\Delta x}{c}$$

Where *c* is the fastest fluid component across all grids or cells. With many spatial cells and a small timestep, the simulation of shockwaves can quickly become computationally expensive. Adaptive mesh refinement allows using varied cell sizes to include fine resolution cells only where needed and dynamic adjustment of cells as the simulation progresses [2]. However, the timestep is still limited by the CFL condition given by the smallest cell size, leading to a highly limited timestep in strong shock simulations.

Meshless methods are a class of approaches that do not rely on a traditional form of spatial discretization for a problem. Unlike mesh-based methods, meshless methods have no fixed connection between discretization nodes that can move throughout the domain. Interactions are computed between neighboring nodes at each timestep. Smoothed Particle Hydrodynamics (SPH) is a meshless method developed by Gingold and Monaghan [3] which uses simulated particles to model a medium. In SPH, fluid properties are approximated at the location of free particles with forces calculated between each neighboring pair. The fluid is represented entirely by the ensemble of particles and advanced through time until the simulation is concluded.

In a typical SPH simulation, the number of interactions per particle per timestep is higher than in grid-based methods. For a regular 2D mesh or particle distribution, SPH particles, on average, interact with 21 particles compared to 9 grid points used in a second-order mesh solver [4]. As a result, SPH simulations are expected to be slower than grid-based methods for most fluid problems. For shock problems, the Lagrangian nature of SPH leads to a natural distribution of the computational expense of the simulation around the shock as the particles clump around it. However, the large number of interactions per particle is expected to outweigh these costs for simulating shocks over a domain with a substantial spatial extent. Regardless, we posit that exploring a hybrid FVM-SPH approach is valuable irrespective of computational speedup due to the possible inclusion of SPH capabilities in many shock applications, including modeling complex geometries, fluid-structure interactions, and deformation and fracture modeling [5].

Various approaches are used to enhance grid-based methods for modeling shockwaves, including higher-order methods, reduced-order methods, and even predictive machine learning [6], [7], [8]. Each approach offers different advantages and disadvantages in accuracy, efficiency, and difficulty of implementation, yet high fidelity simulation of shockwaves remains a significant challenge. This work presents a novel approach using both the cell-based Finite Volume Method and meshless Smoothed Particle Hydrodynamics for shock capturing in compressible fluids. The approach employs the meshless SPH scheme around the shock front while utilizing the efficiency of the cell-based finite volume method for the bulk of the fluid outside of the shock.

## 2 Background

### 2.1 Finite volume method

In the Finite Volume Method (FVM), the domain is discretized into cells or volumes with fluid properties averaged for each enclosed volume. Differential equations for a given system are modeled through the approximation of fluxes across each volume boundary. The FVM employs a structured or unstructured mesh for discretizing problems [9]. The discrete value of a function in each cell is determined from its continuous form:

(2) $$\langle f_{i}\rangle =\frac{1}{\Delta V}\int_{V}f(x)\;dV$$

Where $\langle f_{i}\rangle$ represents the average of *f* for the cell at point *i* with a volume of Δ*V*. Derivative operators in the FVM can be converted into surface fluxes through the divergence theorem. The gradient operator can be expressed as:

(3) $$\langle \nabla f_{i}\rangle =\int_{S}\nabla f(x)\;dS=\sum_{s}{\overset{ˆ}{n}}_{s}{\overset{¯}{f}}_{s}$$

Where ∇ represents the gradient operator, *s* iterates over each surface of a cell volume, ${\overset{ˆ}{n}}_{s}$ is the normal vector for each surface, and ${\overset{¯}{f}}_{s}$ is the flux through each surface.

For compressible fluids, the Euler equations describe the conservation of mass, momentum, and energy. Written generally in conservative form without source terms, they appear as [10]:

(4) $$\frac{\partial \rho }{\partial t}+\nabla \cdot (\rho u)=0,$$

(5) $$\frac{\partial (\rho u)}{\partial t}+\nabla \cdot (\rho u\cdot u)+\nabla p=0,$$

(6) $$\frac{\partial (\rho E)}{\partial t}+\nabla \cdot (\rho Eu+pu)=0,$$

where *ρ* is the density, $u=u\overset{ˆ}{x}+v\overset{ˆ}{y}+w\overset{ˆ}{z}$ is the velocity vector, and *E* is the total energy. *E* can be further broken down into internal energy *e* and kinetic energy:

(7) $$E=e+\frac{|u{|}^{2}}{2}$$

An equation of state is needed to determine *p* and close the system of equations. In this investigation, the gamma-law equation of state is used:

(8) $$e=\frac{p}{\gamma -1}$$

Air, used as the medium for the test problems in this investigation, is largely composed of diatomic molecules with three translational and two rotational degrees of freedom for a total of α=5 degrees of freedom, yielding $\gamma =\frac{\alpha +2}{\alpha }=1.4$.

In one dimensional planar geometry, Equations (4)-(6) become:

(9) $$\frac{\partial \rho }{\partial t}+\frac{\partial (\rho u)}{\partial x}=0$$

(10) $$\frac{\partial (\rho u)}{\partial t}+\frac{\partial (\rho uu+p)}{\partial x}=0$$

(11) $$\frac{\partial (\rho E)}{\partial t}+\frac{\partial (\rho uE+up)}{\partial x}=0$$

Together with the gamma-law equation of state, the FVM can be applied to find the solution to these closed systems of equations for each timestep. Explicit or implicit time integration can be used to advance the simulation until the final result is determined.

### 2.2 Smoothed particle hydrodynamics

Smoothed Particle Hydrodynamics is one of the most common meshless approaches to hydrodynamics. A collection of simulated particles is used to define the field variables and compute interactions needed to evolve the simulation in time. The meshless approach to SPH creates some key differences from mesh-based approaches. SPH must still abide by the CFL condition described in equation (1), but Δ*x* is tethered to a particle's size rather than the size of a mesh cell.

The implementation of boundary conditions in SPH varies significantly from grid-based methods and includes many unique challenges. Periodic and reflective boundaries can be implemented with ghost particles, while inlet and outlet boundaries require careful consideration. [4], [11]. Free surface boundaries, on the other hand, are simpler to implement in SPH than with grid-based methods, as the particles are allowed to naturally form a free boundary with surface particles only influenced by their neighbors, surface tension, and gravity (where applicable) without additional boundary particles needed above the surface [12]. In certain simulations, such as self-gravitating systems in astrophysics, no SPH boundary treatment is needed at all.

In SPH, the domain is discretized through discrete particles whose shape is described by a kernel function $W(x-x^{\prime },h)$, where $x-x^{\prime }$ is the distance from the particle center, and *h* is the characteristic length scale of the kernel, also termed the smoothing length. Functions describing fluid values can be approximated using the kernel function [4]:

(12) $$<f(x)>=\int_{\Omega }f(x^{\prime })W(x-x^{\prime },h)dx^{\prime },$$

where Ω is the volume over which *f* is evaluated. Note that as $W(x-x^{\prime },h)\to \delta (x-x^{\prime })$, equation (12) becomes an identity. Derivatives of functions can be approximated similarly:

(13) $$<\nabla \cdot f(x)>=-\int_{\Omega }f(x^{\prime })\cdot \nabla W(x-x^{\prime },h)dx^{\prime }.$$

For a finite number of particles of a finite size, the functions describing fluid values and the derivatives of fluid values can be written in a discretized form:

(14) $$<f(x_{i})>=\sum_{j}\frac{m_{j}}{\rho_{j}}f(x_{j})W_{ij},$$

(15) $$<\nabla \cdot f(x_{i})>=-\sum_{j}\frac{m_{j}}{\rho_{j}}f(x_{j})\cdot \nabla_{i}W_{ij},$$

where *j* is an index referring to neighbors of a given particle *i*. The spatially discretized integration volume has been inserted using $\Delta V_{j}=\frac{m_{j}}{\rho_{j}}$, and $W_{ij}=W(x_{i}-x_{j},h)$, or the relative strength of interaction between particles. The gradient of $W_{ij}$ is evaluated via:

(16) $$\nabla_{i}W_{ij}=\frac{x_{i}-x_{j}}{r_{ij}}\frac{\partial W_{ij}}{\partial r_{ij}},$$

where $r_{ij}$ is the distance between particle pairs. Note that the fluid functions are evaluated at particle locations. Equation (12) defines the field functions only at particle locations in this formulation. However, field values can be evaluated anywhere by substituting the location of a particle with any arbitrary location.

In SPH, the Euler equations are discretized for each individual particle. Given the Lagrangian nature of SPH, it is helpful to construct the equations in terms of the material derivative. In one dimension, the density of each particle is given by [13]:

(17) $$\rho_{i}=\sum_{j}m_{j}W_{ij},$$

where *j* is an index that refers to all particles that lie within the range of the kernel function $W(x-x^{\prime },h)$. The time evolution of velocity is given by:

(18) $$\frac{du_{i}}{dt}=-\sum_{j}m_{j}(\frac{p_{i}}{\rho_{i}^{2}}+\frac{p_{j}}{\rho_{j}^{2}})\nabla_{i}W_{ij},$$

while the time evolution of particle energy is given by:

(19) $$\frac{de_{i}}{dt}=\frac{1}{2}\sum_{j}m_{j}(\frac{p_{i}}{\rho_{i}^{2}}+\frac{p_{j}}{\rho_{j}^{2}})\cdot \nabla_{i}W_{ij}.$$

Equations (17)-(19) above describe the most basic formulation of SPH and generally will not yield sufficiently accurate results for most problems. As a result, adaptations such as artificial viscosity have been introduced to improve SPH models [14]. The specific approaches used in this investigation are listed in section 3.2.

## 3 Methods

### 3.1 Pyro2

The shock simulations were performed in python using two frameworks to implement a finite volume method and smoothed particle hydrodynamics, respectively. For the FVM implementation, the Pyro2 framework was used [15]. Pyro2 is an open-source code that solves a variety of problems on a two-dimensional mesh. Pyro2 is a strictly finite volume code but provides different solvers for advection, compressible and incompressible hydrodynamics, and thermal diffusion. Several variations for each solver are available, including a piecewise linear method, method of lines, fourth-order Runge-Kutta, and spectral-deferred correction as options for the compressible solvers. The standard piecewise linear compressible solver, which implements the method described in [16], is employed for the initial investigation of the hybrid approach. Standard zero-gradient wall boundaries are employed. The explicit time differencing allows for the more straightforward implementation of coupling and boundary conditions with SPH particles.

The standard compressible Pyro2 solver utilizes a two-dimensional NumPy array to store conservative mesh variables, including density, internal energy, and x- and y- momenta, and has built-in conversion functions for finding primitive variables: density, pressure, and x- and y- velocities. The python package Numba [17] accelerates calculations and improves performance in the Pyro2 backend for evolving the simulation in time. As an explicit FVM code written in Python, Pyro2 is less efficient than alternatives employing compiled software. However, the software architecture and numerous solvers and methods available in Pyro2 offer flexibility and rapid iteration of the novel FVM-SPH coupling mechanisms presented in this work.

### 3.2 PySPH

PySPH is an open-source python framework for implementing SPH methods [18]. PySPH has several formulations available, including compressible and incompressible SPH, Weakly Compressible SPH, Adaptive Density Kernel Estimation (ADKE), Godunov SPH (GSPH), and Conservative Reproducible Kernel SPH. There are also SPH approaches included for elastic simulations, various boundary methods, corrective SPH methods, and surface tension methods. This investigation uses the ADKE and GSPH schemes for the one-dimensional shock problems. Reflecting wall boundaries are used in each case.

PySPH contains custom data structures for particle arrays compatible with NumPy and accelerated through Cython [19] at runtime. The framework computes particle interactions at each timestep through several available nearest neighbor particle search (NNPS) algorithms. In these one-dimensional test problems, a linked-list NNPS algorithm is employed. Various kernel functions describe particle shape and determine the strength of relative particle interactions. A Gaussian kernel is used in the one-dimensional test problems given by [4]:

(20) $$W(x-x^{\prime },h)=\frac{e^{{\left(\frac{\left|x-x^{\prime }\right|}{h}\right)}^{2}}}{h\sqrt{\pi }}$$

Where $|x-x^{\prime }|<3$. If $|x-x^{\prime }|\geq 3$, then W→0.

The ADKE scheme is one of the two schemes investigated for one-dimensional test problems. Monaghan and Gingold [14] were the first to successfully apply SPH to shock problems through the introduction of artificial viscosity terms. Subsequently, a viscous pressure term was added to the momentum equation to prevent particles from streaming past one another and generating unphysical results, and a dissipative term was added to the energy equation to prevent an unphysical spike in pressure at the discontinuity [20]. The equations used in PySPH for the ADKE formulation are listed in the appendix.

The GSPH scheme utilizes a Riemann solver for accurate solutions to shock problems. Riemann solvers have been used extensively in grid-based methods for resolving shocks. The Godunov scheme uses the solution to the Riemann problem for every cell interface to find the numerical flux between each cell. The GSPH scheme incorporates the same principle but instead uses the solution to the Riemann problem for every particle pair [21]. The implementation of the scheme in PySPH also includes a variable smoothing length to alter the spatial resolution of the simulation where needed. The equations used in the GSPH scheme are listed in the appendix.

## 4 FVM-SPH coupling

SPH simulations are, in general, less efficient than mesh-based simulations because the number of particle-pair interactions that must be calculated is greater than the number of neighbors needed for each mesh cell calculation. In shock problems, a large number of particle interactions are computed for particles that are located well past the interaction range of the shockwave. The spatial resolution provided by these particles is unnecessary until the shockwave travels within range. By incorporating the SPH method as a type of subgrid modeling on a finite volume mesh, the SPH particles are only included around the regions of discontinuous fluid. Additionally, since the discontinuous regions around shocks are simulated with SPH, a coarser FVM grid can be used to achieve the same resolution as a finer FVM without SPH particles.

SPH has previously been used in conjunction with other methods to realize the unique benefits of SPH without its disadvantages. A common application of hybrid SPH approaches is the modeling of fluid-structure interactions. SPH is very capable of modeling deformable or fracturing solids and, as a result, is typically used for structures in hybrid fluid-structure interaction simulations [22], [23], [24]. SPH has also previously been coupled with FVM to simulate incompressible fluids [25], [26], [27]. SPH is typically used to simulate the boundary layer or a free surface, while FVM cells are used for bulk regions of fluids. Coupling is done using ghost particles and cells or hybrid weighting zones for passing information between the SPH and FVM regions. To our knowledge, no previous research demonstrates the application of a hybrid SPH-FVM approach to a compressible fluid.

**Figure 1:**
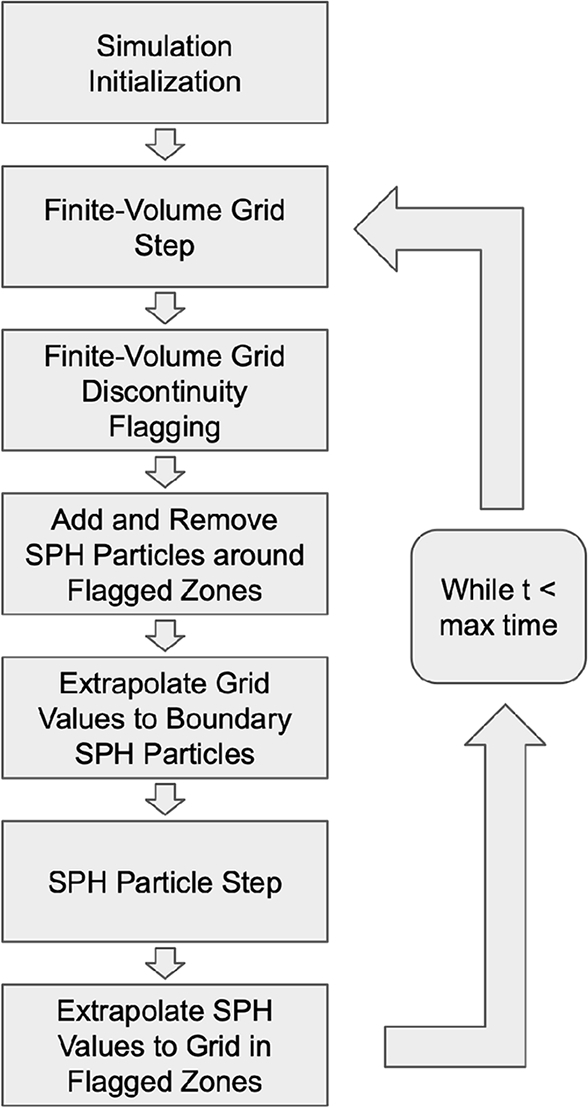
Diagram of the sequential steps taken by the FVM-SPH coupling algorithm.

The steps used in the novel FVM-SPH algorithm are displayed in Fig. 1. The FVM is used as the main simulation, while the SPH method is used as a subgrid model on the mesh where needed. The Pyro2 simulation is initialized first and the grid is populated with the initial fluid values from the problem specification. The PySPH simulation is then initialized with particles only introduced near the initial discontinuities.

A timestep begins by advancing the FVM mesh forward one step in time. Pyro2 allows for an adaptive timestep size, but for simplicity in coupling with SPH, a fixed timestep is used in each problem. After every timestep, the FVM mesh is scanned for discontinuities by calculating the derivative of each variable and flagging regions with a derivative greater than a predetermined threshold. Two sets of zones are defined by the location of flagged discontinuous regions: an active particle zone, where SPH particles will be used to model the local fluid, and ghost particle zones, where SPH ghost particles are placed to serve as boundary particles for the active SPH particles.

The fluid values in the active particle zone are interpolated to the overlaying FVM mesh at every timestep using linear approximation and serve as boundary cells to the adjacent FVM cells. Similarly, the values of the FVM cells in the ghost particle regions are interpolated to the SPH ghost particles at every timestep. The boundary layers are set to a value greater than the interaction distance for a given cell or particle. The interaction distance Δ is defined as the distance at which $W(x-x^{\prime },h)$ goes to zero for $\Delta >x-x^{\prime }$, and Δ>n⁎dx for the FVM mesh. *dx* is the distance between mesh cells and *n* is the number of cells needed for the calculation of updated values for a single cell for the FVM solver used.

Once the SPH particle regions have been defined for a given timestep, SPH particles are injected where absent in flagged regions and tagged as ghost particles if they lie within the ghost particle regions. Since particle interaction is weighted by the smoothing length *h*, particles are only injected near existing particles with a regular spacing proportional to *h*, defined at the initialization of the SPH simulation. SPH particles are also deleted in regions that no longer lie within ghost particle regions as the simulation advances.

Extrapolating values between particles and the FVM mesh requires interpolating fluid values in the appropriate regions. When the buffer size, used to calculate the active particle and ghost particle regions in discontinuous areas, is larger than the interaction distance, linear interpolation is sufficient for accurate results. For a given ghost particle or cell, the value of a field *f* at the location $x_{i}$ is given by:

(21) $$f(x_{i})=\frac{f(x_{l})(x_{r}-x_{i})+f(x_{r})(x_{i}-x_{l})}{x_{r}-x_{l}}$$

Where $x_{l}$ and $x_{r}$ are the locations of the nearest particles or cell centers to the left and right of $x_{i}$. Using an interpolation scheme introduces a source of non-conservation for the typically conservative FVM approach. Still the resulting error in the problems examined in this study was not found to be significant. Once values are interpolated from the FVM mesh to new active SPH particles and all ghost SPH particles, the SPH particles are advanced forward one timestep, equal to the timestep defined in the FVM simulation. The FVM and SPH schemes are alternatively advanced forward in time until the final time is reached.

Special care must be taken in ghost particle regions to ensure the stability of the SPH simulation. The smoothing length *h* is held constant for ghost particles to avoid having to calculate *h* at every timestep. As a consequence, the spacing between the ghost particles and active particles must remain regular for the updated active particle values to be calculated with the appropriate boundary values. The ghost particles are repositioned at each timestep such that the spacing of particles adjacent to the active particles is regular before the FVM cell values are interpolated to the ghost particles.

## 5 Results

The simulations were performed using Oregon State University College of Engineering's HPC platform Rogue in a serial configuration. The code was built using a conda virtual environment with all the respective python dependencies for Pyro2 and PySPH installed, allowing each framework to run independently or as elements of the hybrid code. For each test problem, the simulation is first performed using both Pyro2 and PySPH independently to establish a baseline timing comparison; then, the hybrid implementation is performed and compared.

### 5.1 Sod shock tube

The Sod problem was first developed in 1978 and involves a left/right discontinuity [28]. The initial conditions are given by ρ(left,right)=(1.0,0.125), p(left,right)=(1.0,0.1), and u=0. The timestep is set to 1e-4 and the maximum time of the simulation is set to t=0.2. The length of the domain is equal to 1.0, and the discontinuity is placed in the center. Three waves result from the initial discontinuity: a rarefaction wave on the left, a shock on the right, and a density discontinuity between the two and traveling to the right. The ADKE scheme is used in PySPH to model the Sod shock tube problem.

**Figure 2:**
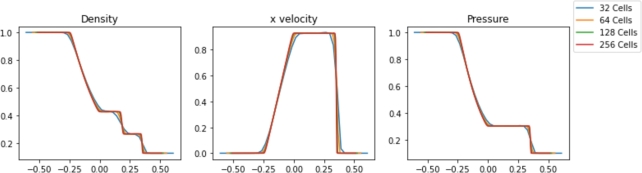
Pyro2 results from the Sod shock tube problem at various resolutions at t=0.2.

**Figure 3:**
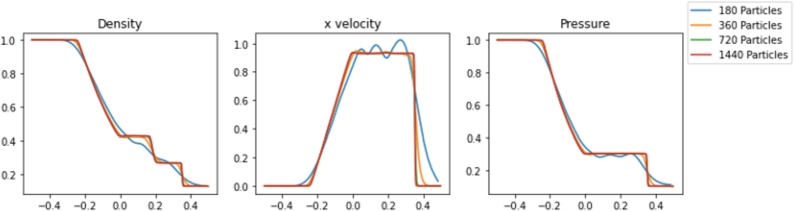
PySPH results from the Sod shock tube problem at various resolutions at t=0.2.

Simulations were performed in Pyro2 and PySPH for a range of resolutions. The results are shown in Figure 2, Figure 3, respectively. The Sod problem is not a challenging problem for traditional mesh-based approaches, and Pyro2 is able to accurately model the results with only 128 horizontal cells and one vertical cell. 720 PySPH particles are required to resolve the shockwaves without blurring, while instabilities begin to arise in simulations with 180 particles or less. The simulation runtimes for each case are displayed in Table 1. Also included is L2 Norm relative to the exact solution for each case and the smoothing length used in each PySPH simulation. In absolute terms, both Pyro2 and PySPH were able to efficiently model the problem, with both approaches only requiring less than 1 and 10 seconds to run, respectively. Reducing the resolution in PySPH did not result in a significant reduction in simulation time, likely due to the computational overhead of initializing the simulations.

**Table 1:** Pyro2 and PySPH simulation runtimes and L2 Norm relative to the exact solution for the Sod shock tube problem for various resolutions.

Pyro2	Resolution	256 Cells	128 Cells	64 Cells	32 Cells
	Runtime	2.20s	0.965s	0.293s	0.110s
	L2 Norm	0.07779	0.09994	0.1268	0.1378



PySPH	Resolution	1440 Particles	720 Particles	360 Particles	180 Particles
	h	1.2	1.2	1.2	1.2
	Runtime	9.67s	9.30s	9.04s	10.37s
	L2 Norm	0.1910	0.1968	0.2734	0.4388

**Figure 4:**
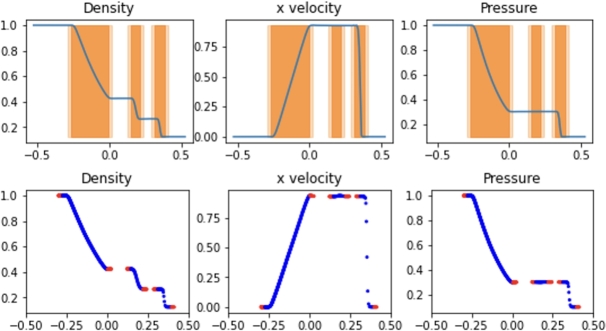
Hybrid SPH results from the Sod shock tube problem at t=0.2. Active SPH regions are highlighted in dark orange while SPH ghost regions are highlighted in light orange. Blue and red particles depict active and ghost SPH particles, respectively.

The results for the hybrid implementation are displayed in Fig. 4. The optimal parameters used in the hybrid implementation for efficiency and accuracy included a flagging threshold of 3.0, meaning a spatial gradient of any of the field variables greater than 3.0 resulted in the cell being flagged for SPH treatment. Greater threshold values resulted in curved field lines at the interfaces of the FVM and SPH regions which resulted in reduced accuracy, while smaller threshold values increased runtime without improving accuracy. A ghost distance of 0.05 was used, meaning cells up to 0.05 away from flagged cells were marked for ghost SPH particles. The active SPH distance, flagging cells for active SPH particles, is set to half that amount. This distance created a boundary layer of more than 5 particles for any SPH boundary region for the entire simulation, which was found to ensure active SPH particles observe proper boundaries.

The upper plot in 4 shows the final solution on the FVM grid with highlighted regions depicting the active SPH particle regions in dark orange and ghost SPH particle regions in light orange. The lower plot displays the location and values of SPH particles, with active particles highlighted in blue and ghost particles highlighted in red. The values of the cells in the dark orange regions are determined by the blue SPH particles at every step, while the values at the locations of the red particles are determined by the cells in the light orange regions. With the optimal hybrid parameters used by the simulation in Fig. 4, the simulation runtime was 17.2s, which is greater than the runtime for any independent Pyro2 or PySPH Sod simulation. The L2 Norm of the approach was 0.1928. However, the hybrid approach results in approximately the same accuracy as the 1440 or 720 particle SPH simulations when using only 32 FVM cells coupled with SPH. The SPH resolution corresponded to the particle density of the 720-particle PySPH simulation. At the final time t=0.2, a total of 302 SPH particles are included in the simulation, along with 40 FVM cells when including ghost cells at the edges of the domain.

### 5.2 Strong shock problem

The strong shock test problem emulates the Sod shock tube problem but with a uniform density and a significantly larger pressure difference. The initial conditions are as follows: ρ=1.0, u=0, and p(left,right)=(1000,0.01). The timestep is set to 5e-6 and the maximum time of the simulation is set to t=0.012. The enormous pressure difference causes a large shock with a Mach number of 198 to form with a rarefaction wave traveling in the opposite direction [29]. The GSPH scheme is used in PySPH to model the strong shock problem.

**Figure 5:**
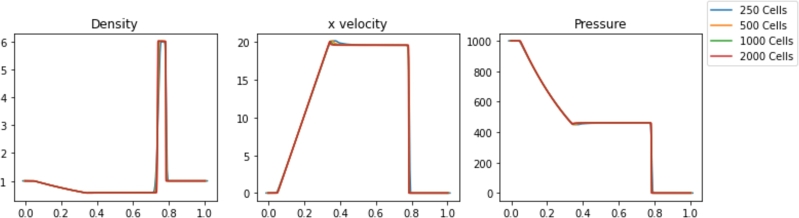
Pyro2 results from the strong shock problem at various resolutions at t=0.012.

**Figure 6:**
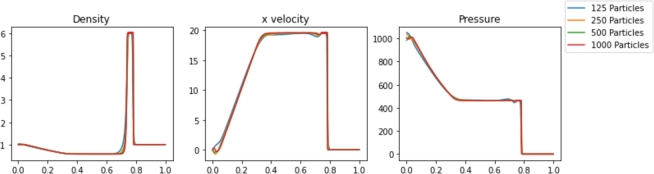
PySPH results from the strong shock problem at various resolutions at t=0.012.

The results for various Pyro2 and PySPH resolutions are shown in Figure 5, Figure 6, respectively. The powerful shockwave results in an almost square density wave with a peak density of approximately 6. Pyro2 and PySPH can reasonably resolve the shock wave with the range of resolutions presented, but higher resolutions of 1000 cells or 500 particles are needed to capture the square shape of the density wave. The timing of the results from the shock wave simulations is shown in Table 2. Also included is L2 Norm for each case relative to a converged solution and the smoothing length used in each PySPH simulation. With the GSPH formulation used in PySPH, the SPH simulations are faster than the FVM simulations for a given resolution required to resolve the shock. As Pyro2 is a strictly 2D FVM code, the poor performance relative to PySPH can be partly explained by the required calculations and boundary layers modeled in the transverse dimension.

**Table 2:** Pyro2 and PySPH simulation runtimes and L2 Norm relative to a converged solution for the strong shock problem for various resolutions.

Pyro2	Resolution	2000 Cells	1000 Cells	500 Cells	250 Cells
	Runtime	222s	117s	61.8s	36.4s
	L2 Norm	1.849	2.611	4.155	4.406



PySPH	Resolution	1000 Particles	500 Particles	250 Particles	125 Particles
	h	1.5	1.5	1.5	1.5
	Runtime	30.1s	28.4s	20.9s	16.8s
	L2 Norm	2.262	2.749	4.343	4.675

**Figure 7:**
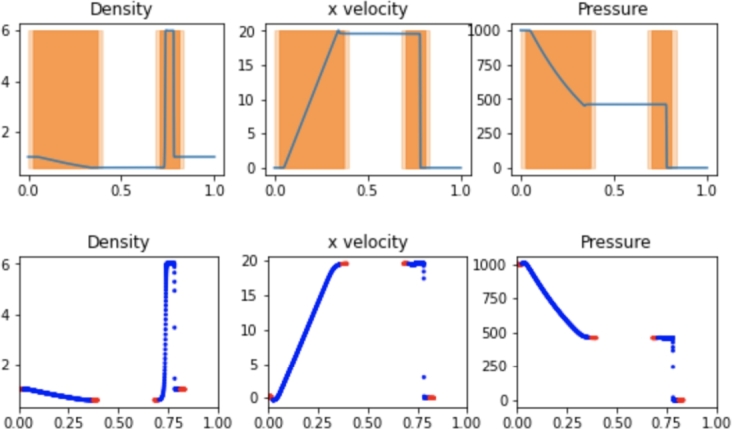
Hybrid SPH results from the strong shock problem at t=0.012. Active SPH regions are highlighted in dark orange while SPH ghost regions are highlighted in light orange. Blue and red particles depict active and ghost SPH particles, respectively.

The results of the hybrid shock simulation are shown in Fig. 7. The hybrid implementation leads to a similar runtime as the simulation using only PySPH with 29.7s. The L2 Norm relative to a converged solution of the approach was 2.169. The optimal parameters used in the hybrid implementation for efficiency and accuracy included a gradient flagging threshold of 2000. The greater threshold value is a result of the larger gradients in the shock problem, but as a relative parameter it is primarily useful for partitioning the problem in FVM and SPH zones and the larger value does not impact accuracy. Lower thresholds led to reduced computational efficiency as the vast majority of the domain becomes an active SPH simulation with the overhead of the hybrid implementation. A ghost distance of 0.05 was used with the active SPH distance being half that amount. The optimal hybrid simulation allowed for the use of 250 FVM cells and a 1000 SPH particle resolution. With any lower FVM or SPH resolution, the accuracy of the hybrid implementation decreases. At the final time t=0.012, a total of 672 SPH particles are included in the simulation, along with 258 FVM cells when including ghost cells at the edges of the domain.

### 5.3 Woodward-Colella blastwave

The Woodward-Collela blastwave is a problem designed to test the limits of a computational fluid dynamics code. The problem consists of two shock waves of different strengths originating on either side of a simulated domain and colliding and interacting in the center [30]. The initial conditions are given by: ρ=1.0, u=0, and p(1,2,3)=(1000,0.01,100) where regions 1 and 3 are defined by x<0.1 and x>0.9, respectively, with region 2 defined between regions 1 and 3. The timestep is set to 5e-6 with the maximum time set to t=0.038. High resolution is required to resolve the interaction of the shockwaves at each timestep. The GSPH scheme is used in PySPH to model the strong shock problem.

**Figure 8:**
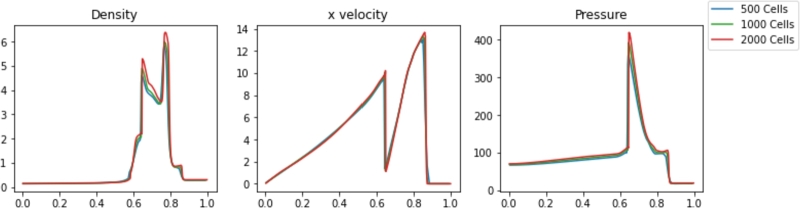
Pyro2 results from the Woodward-Colella blastwave at various resolutions at t=0.038.

**Figure 9:**
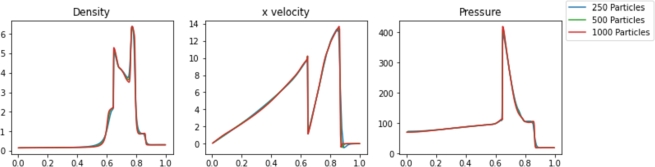
PySPH results from the Woodward-Colella blastwave at various resolutions at t=0.038.

The results for various Pyro2 and PySPH resolutions are shown in Figure 8, Figure 9, respectively. High resolution is needed in both simulations to resolve the accurate solutions in each case. 2000 FVM cells are required to accurately resolve the shape of the interacting blastwaves at the final time. The 1000 and 500 particle SPH simulations are both able to resolve the features of the shocks, but with degraded resolution around some of the sharp edges. The timing of the results from the Woodward-Colella blastwave simulations is shown in Table 3. Also included is L2 Norm relative to a converged solution for each case and the smoothing length used in each PySPH simulation. The SPH simulations appear much faster than the FVM simulations at a given resolution, but as before, the modeling of the unnecessary transverse dimension in Pyro2 decreases performance relative to PySPH.

**Table 3:** Pyro2 and PySPH simulation runtimes and L2 Norm relative to a converged solution for the Woodward-Colella blastwave for various resolutions at t=0.038.

Pyro2	Resolution	2000 Cells	1000 Cells	500 Cells
	Runtime	697s	376s	205s
	L2 Norm	4.735	10.977	13.145



PySPH	Resolution	1000 Particles	500 Particles	250 Particles
	h	1.5	1.5	1.5
	Runtime	96.2s	71.8s	59.0s
	L2 Norm	2.532	3.238	4.921

**Figure 10:**
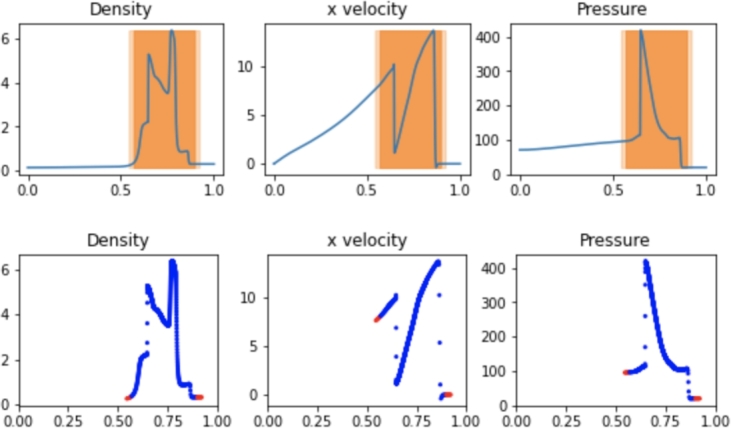
Hybrid SPH results from the Woodward-Collela blastwave. Active SPH regions are highlighted in dark orange while SPH ghost regions are highlighted in light orange. Blue and red particles depict active and ghost SPH particles, respectively.

The results of the hybrid shock simulation are shown in Fig. 10. The hybrid implementation leads to a slightly faster runtime compared to the PySPH implementation with 84.3s. The L2 Norm of the approach was 2.550. This is largely because a low-resolution FVM mesh can be used on a fraction of the simulated domain while fewer SPH particles are needed to achieve results with the same accuracy. The optimal parameters used in the hybrid implementation for efficiency and accuracy included a flagging threshold of 2000, a ghost distance of 0.05, and an active SPH distance half of that value. The optimal hybrid simulation allowed for the use of 250 FVM cells and a 1000 SPH particle resolution. At the final time t=0.038, a total of 784 SPH particles are included in the simulation, along with 508 FVM cells when including ghost cells at the edges of the domain.

### 5.4 FVM-SPH domain partition

The hybrid algorithm flags discontinuities in the field variables using a sensitivity variable called the discontinuity threshold. The threshold value is a relative term that is used to partition the space with the goal of efficiently dividing the domain between the FVM mesh and SPH particles. For problems with stronger shocks, more of the domain will become dominated by regions with high field gradients, and more of the domain will be flagged for SPH treatment for a given threshold value. However, because the discretization size varies between problems, the threshold value is not directly correlated with the accuracy of the final solution in hybrid simulations.

For each hybrid simulation, an appropriate threshold must be selected to balance the accuracy and efficiency. A low threshold value will result in more of the domain being flagged and populated with SPH particles, increasing the computational expense by favoring SPH over the computationally cheaper FVM mesh, particularly when a coarse mesh is used. A high threshold value will result in field gradients at the site of FVM-SPH boundaries, which can negatively impact the accuracy of the coupling scheme used, particularly for boundary SPH particles which are highly sensitive to the value and position of neighboring ghost particles.

**Figure 11:**
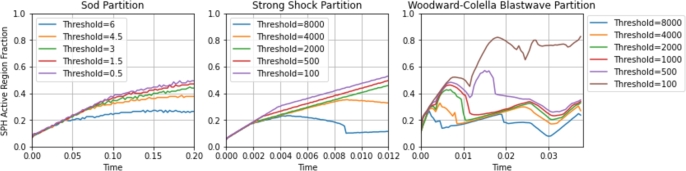
FVM-SPH active simulation partition for each test problem for a number of discontinuity thresholds over the duration of the simulation.

For a given problem, a threshold is selected by conducting a scan over a range of options and determining the optimal threshold for efficiency without reducing accuracy. A threshold of 3 was found to be optimal for the Sod shock tube problem, while thresholds of 2000 were used for the strong shock and Woodward-Colella problems. A range of thresholds was investigated in each case, and plots showing the partition of FVM and SPH active simulation are shown in Fig. 11. A higher threshold leads to an increased use of SPH in a simulation, but the partition ultimately depends on the nature of the problem. In the Sod problem, the partition grows steadily for each threshold as the pressure and density shocks diverge and spread. At a threshold of 8000, the strong shock partition sees a large drop as the gradient of the rarefaction wave shrinks below the threshold. The interacting shocks in the Woodward-Colella problem result in a highly variable partition over time as shocks collide, diverge, and reflect.

The optimal threshold values typically correspond to the strength of the largest shock, and this can be used to estimate the threshold for similar problems. For example, the strength of the largest shock in the Woodward-Colella problem is the same as the strength of the shock in the strong shock problem, and so the same threshold was used and found to be approximately optimal in each case. In further investigations, a table of thresholds for a given shock strength or an empirical relationship can be used to streamline hybrid simulations. However, complex problems that include features such as colliding shocks or vacuums should be thoroughly investigated to determine the relationship between the threshold and the accuracy and efficiency.

## 6 Discussion

The cost of coupling the two approaches together will lead to an increase in runtime unless each approach has advantages in different parts of the domain. The hybrid approach for the Sod problem leads to a longer runtime when compared with the FVM and SPH approaches alone since the FVM approach can easily model the problem. Even the SPH approach, while less efficient, is faster than the hybrid approach due to coupling overhead in the Sod problem. In the strong shock problem and Woodward-Colella blastwave, the advantages of each approach captured by the hybrid method outweigh the overhead of coupling the Pyro2 and PySPH simulation codes. We cannot conclude that coupling a more efficient, compiled FVM solver with SPH will produce better results than the FVM solver alone. Still, it does indicate that coupling the FVM and SPH while preserving accuracy and efficiency is possible.

One natural advantage of implementing a hybrid method is that the approach allows for a form of multi-resolution modeling to be included between the two methods. Different orders of resolution can be used in the FVM and SPH methods meaning a coarser resolution can be used in parts of the domain. Multi-resolution modeling is common in hydrodynamics, with AMR being a dynamic form of a multi-resolution approach. While additional effort is needed to include multi-resolution modeling in a standard approach, the multi-resolution functionality is already built into the hybrid SPH method, as the interpolative coupling works for a range of different resolutions. If the resolution becomes too low, however, the size of boundary cells may inhibit the accurate and efficient simulation of the problem.

A unique advantage of the hybrid approach is the Lagrangian characteristics of SPH are retained while reducing the total range of SPH particles. As the shockwaves move through the simulation, the SPH particles are swept along and clump up at the location of the shocks, providing a higher resolution at the shock front. In the strong shock problem, the SPH number density varies by a factor of 10 across the extremes in the domain, while in the Woodward-Colella blastwave, the number density varies by over 30. The high density around shocks allows for simulations with a low number of SPH particles globally to have surprisingly good resolution, as is seen in the 500-particle Woodward-Colella simulation, which only required 72 seconds of runtime. Through coupling FVM and SPH, the number of cells and particles to attain a high resolution can be minimized, leading to an increase in computational efficiency.

An interesting feature of the SPH approach is that the simulation runtime doesn't scale with the resolution. One aspect of this behavior is likely due to the fixed computational costs of the simulation, but initialization of the problem until the first timestep begins only takes approximately 0.1 seconds in each case. A larger contributor is likely the Lagrangian nature of the method and the resulting clumps of particles around shocks being sizable regardless of the initial resolution. This effect is presumably responsible for some of the efficiency of the hybrid method as increasing the resolution of SPH used, and therefore the accuracy of the final result, only marginally increases computational cost.

## 7 Conclusion

We have developed a new shock-capturing method coupling the Finite Volume Method framework Pyro2 with Smoothed Particle Hydrodynamics framework PySPH and investigated its application to 1D shock problems. The method uses a coarse FVM mesh across the simulation domain, while SPH particles are used around the location of discontinuities. Active SPH particles in zones around discontinuities interpolate values to adjacent FVM cells at every timestep, with SPH ghost particles bordering the SPH active particles and drawing values from adjacent FVM cells every timestep. The SPH active zones are adjusted each timestep as the shock travels through the simulation with SPH particles added or deleted in newly activated or deactivated particle regions. The location of ghost particles is set to be spaced at a regular distance from active SPH particles to ensure proper boundary treatment.

The hybrid approach shows potential in accurately and efficiently modeling strong shock problems. SPH schemes accurately resolve the shock waves, while the FVM grid limits the number of SPH particles needed across the domain. The hybrid implementation adds computational overhead, but the gains in efficiency outweigh the cost for the strong shock and Woodward-Colella blastwave problems. Furthermore, the coupling of FVM and SPH for shock problems allows for the future inclusion of SPH capabilities, such modeling complex geometries and fluid-structure interaction with large deformations or fracturing.

The demonstrated advantage of the hybrid approach for strong shocks in one dimension is expected to increase in two dimensional problems, where the number of particles or cells is increased by the square of the resolution. The number of SPH particle interactions also increases significantly for each additional dimension, but coupling the approach to a coarse FVM for 2D shock problems may result in a significant reduction in baseline computational costs. The extension of the hybrid method to two dimensions will be presented in future work, along with the results for a number of two-dimensional test problems.

## CRediT authorship contribution statement

Conner Myers: Conceived and designed the experiments; Performed the experiments; Analyzed and interpreted the data; Wrote the paper.

Camille Palmer, Todd Palmer: Conceived and designed the experiments; Analyzed and interpreted the data.

## Declaration of Competing Interest

The authors declare the following financial interests/personal relationships which may be considered as potential competing interests: Conner Myers reports financial support, article publishing charges, and travel were provided by 10.13039/100006234Sandia National Laboratories under contract number 2214652.

## Data Availability

Data associated with this study has been deposited at https://github.com/myerscon/hybridSPH/.

## Appendix A SPH formulations

### A.1 ADKE formulation

The ADKE formulation implemented in PySPH is given by [20]. The density equation becomes:

(A.1) $$\rho_{i}=\sum_{j}m_{j}{\overset{˜}{W}}_{ij}$$

Where ${\overset{˜}{W}}_{ij}$ is the symmetrized weighting function between a particle pair given by ${\overset{˜}{W}}_{ij}=\frac{W_{ij}+W_{ji}}{2}$. The momentum equation is given by:

(A.2) $$\frac{dv_{i}}{dt}=-\sum_{j}m_{j}(\frac{p_{i}}{{\rho_{i}}^{2}}+\frac{p_{j}}{{\rho_{j}}^{2}}+\Pi_{ij})\nabla_{i}{\overset{˜}{W}}_{ij}$$

Where $\Pi_{ij}$ is an artificial viscosity term given by:

(A.3) $$\Pi_{ij}=\frac{-\alpha {\overset{˜}{c}}_{ij}\mu_{ij}+\beta {\mu_{ij}}^{2}}{{\overset{˜}{\rho }}_{ij}}$$

With

(A.4) $$\mu_{ij}=\frac{h_{ij}v_{ij}\cdot x_{ij}}{|x_{ij}{|}^{2}+\eta^{2}}$$

Where $\mu_{ij}$ for $v_{ij}\cdot x_{ij}<0$, so that the term is only nonzero for approaching pairs of particles. *α*, *β*, and *η* are numerical terms set at the start of the simulation, and ${\overset{˜}{c}}_{ij}$ and ${\overset{˜}{\rho }}_{ij}$ are the averaged sound speed and density between the two particles, respectively.

The energy equation is given by:

(A.5) $$\frac{de_{i}}{dt}=\frac{1}{2}\sum_{j}m_{j}[(\frac{p_{i}}{{\rho_{i}}^{2}}+\frac{p_{j}}{{\rho_{j}}^{2}}+\Pi_{ij})v_{ij}+2H_{ij}x_{ij}]\cdot \nabla {\overset{˜}{W}}_{ij}$$

The added heating term $H_{ij}$ is given by:

(A.6) $$H_{ij}=\frac{\left(H_{i}+H_{j})(e_{i}-e_{j}\right)}{{\overset{˜}{\rho }}_{ij}|x_{ij}{|}^{2}+\eta^{2}}$$

With the thermal conduction coefficient H defined by:

(A.7) $$H_{i}=g_{1}h_{i}c_{i}+g_{2}{h_{i}}^{2}[|(\nabla \cdot v_{i})|-(\nabla \cdot v_{i})]$$

With the numerical parameters $g_{1}$ and $g_{2}$ set at the start of the population. The heating term vanishes for $\nabla \cdot v_{i}\geq 0$. The adaptive density is the ADKE formulation is done by first estimating *ρ* for each particle through equation (A.1) using $h=h_{0}$ where $h_{0}$ is the initial smoothing length, determined by the particle separation at the start of the simulation. The estimation then follows:

(A.8) $$log(g)=\frac{1}{N}\sum_{j=1}^{N}log({\overset{˜}{\rho }}_{j})$$

(A.9) $$\lambda_{i}=k{\left(\frac{g}{{\overset{˜}{\rho }}_{i}}\right)}^{\epsilon }$$

And lastly

(A.10) $$h_{i}=\lambda_{i}h_{0}$$

The parameters *k* and *ϵ* are tuning parameters set before the simulation. For the choices of parameters k=1, ϵ=0, $g_{1}=0$, and $g_{2}=0$, the ADKE formulation collapses to the standard SPH formulation.

### A.2 GSPH formulation

The ADKE formulation implemented in PySPH is given by [21]. The standard or symmetrized density equations can be used, given by equation (17) and (A.1), respectively. The GSPH scheme utilizes solutions to the Riemann problem, treating two adjacent particles as the left and right states of an initial discontinuity. The energy and momentum equations are given by:

(A.11) $$\frac{dv_{i}}{dt}=-\sum_{j}m_{j}p_{ij}^{⁎}[{V_{ij}}^{2}(h_{i})\nabla W_{ij}(\sqrt{2}h_{i})+{V_{ij}}^{2}(h_{j})\nabla W_{ij}(\sqrt{2}h_{j})]$$

(A.12) $$\frac{de_{i}}{dt}=-\sum_{j}m_{j}p_{ij}^{⁎}[v_{ij}^{⁎}-{\overset{˙}{x}}_{i}]\cdot [{V_{ij}}^{2}(h_{i})\nabla W_{ij}(\sqrt{2}h_{i})+{V_{ij}}^{2}(h_{j})\nabla W_{ij}(\sqrt{2}h_{j})]$$

$V_{ij}$ is dependent on the interpolation scheme used for values between particles. The starred quantities are intermediate states determined by the solution to the Riemann problem for each particle pair. The above equations are only valid in the case of a Gaussian kernel. For other types of kernels approximated forms can be used as derived in [31]. The momentum equation can be approximated by:

(A.13) $$\frac{dv_{i}}{dt}=-\sum_{j}m_{j}p_{ij}^{⁎}(\frac{\nabla {\overset{˜}{W}}_{ij}}{\rho_{i}^{2}}+\frac{\nabla {\overset{˜}{W}}_{ij}}{\rho_{j}^{2}})$$

With the energy equation being:

(A.14) $$\frac{de_{i}}{dt}=-\sum_{j}m_{j}p_{ij}^{⁎}[v_{i}-{\overset{˙}{x}}_{i}](\frac{\nabla {\overset{˜}{W}}_{ij}}{\rho_{i}^{2}}+\frac{\nabla {\overset{˜}{W}}_{ij}}{\rho_{j}^{2}})$$

Variable smoothing lengths in the GSPH scheme are determined by:

(A.15) $$\overset{ˆ}{\rho_{i}}=\sum_{j}m_{j}{\overset{˜}{W}}_{ij}(C_{smooth},h_{i})$$

Where $\overset{ˆ}{\rho_{i}}$ is a smoothed density and $C_{Smooth}$ is a numerical smoothing parameter set at the start of the simulation. Using the smoothed density, the smoothing length is then determined through:

(A.16) $$h_{i}=\eta {\left(\frac{m_{i}}{\overset{ˆ}{\rho_{i}}}\right)}^{\frac{1}{d}}$$
